# The Fruits of *Siraitia grosvenorii*: A Review of a Chinese Food-Medicine

**DOI:** 10.3389/fphar.2019.01400

**Published:** 2019-11-22

**Authors:** Xue Gong, Namuhan Chen, Kai Ren, Junying Jia, Kunhua Wei, Le Zhang, Ying Lv, Jianhua Wang, Minhui Li

**Affiliations:** ^1^Department of Pharmacy, Baotou Medical College, Baotou, China; ^2^Guangxi Key Laboratory of Medicinal Resources Protection and Genetic Improvement, Guangxi Botanical Garden of Medicinal Plants, Nanning, China; ^3^Agricultural College, Inner Mongolia University for Nationalities, Tongliao, China; ^4^Pharmaceutical Laboratory, Inner Mongolia Autonomous Region Academy of Chinese Medicine, Hohhot, China; ^5^Department of Technology, Chifeng Institute for Drug Control, Chifeng, China; ^6^Department of Pharmacy, Inner Mongolia Medical University, Hohhot, China; ^7^Inner Mongolia Key Laboratory of Characteristic Geoherbs Resources Protection and Utilization, Baotou Medical College, Baotou, China

**Keywords:** *Siraitia grosvenorii*, ethnopharmacology, food and nutritional value, chemical constituent, antioxidant

## Abstract

*Siraitia grosvenorii* (Swingle) C. Jeffrey, a member of the family Cucurbitaceae, is a unique economic and medicinal plant grown in China. For more than 300 years, *S. grosvenorii* has been used as a natural sweetener and as a traditional medicine for the treatment of pharyngitis, pharyngeal pain, as well as an anti-tussive remedy in China. It is one of the first approved medicine food homology species in China. It has been widely studied as a natural product with high development potential. Therefore, the present paper provides a review of the botanical characterization, traditional uses and ethnopharmacology, food and nutritional values, chemical constituents, pharmacological effects, toxicology, and development direction for the future of *S. grosvenorii*. Phytochemical studies have revealed that the chemical composition of this plant mainly includes iridoid and phenylpropanoid glycosides. Several compounds such as triterpenoids, flavonoids, and amino acids have been isolated from the plant. *S. grosvenorii* and its active constituents possess broad pharmacological properties, such as antioxidant, hypoglycemic, immunologic, anti-tussive and sputum-reducing, hepatoprotective, and antimicrobial activities, etc. By documenting the comprehensive information of *S. grosvenorii*, we hope to establishes the groundwork for further research on the mechanism of action of *S. grosvenorii* and its development as a new health food in the future.

## Introduction

*Siraitia grosvenorii* (Swingle) C. Jeffrey is a species of the genus *Siraitia* Merr. (Cucurbitaceae) and is native to the southern parts of China, mainly the Guangxi Province (Lu and Zhang, 1984). There is a total of seven species belonging to this genus; four of them are native to China, of which *S. grosvenorii* and *Siraitia siamensis* (Craib) C. Jeffrey ex Zhong et D. Fang have medicinal value (Chen Y et al., 2006).The formal Chinese name of *S. grosvenorii* is *luo han guo* (Chinese: 罗汉果), and it is locally known as *lahanguo*, *jiakugua*, *changshouguo*, or *guangguomubie* (Lu and Zhang, 1984).

*S. grosvenorii* is used not only as a food ingredient but also as an herbal medicine, particularly in China, where it has been used as a natural antitussive and expectorant for 300 years (Lu and Zhang, 1984). Furthermore, it is one of the first approved medicine food homology (MFH) species in China (The concept of “medicine and food homology” was mentioned in the Huang Di Nei Jing Su Wen: “eating on an empty stomach as food and administering to the patient as medication, ” which reflects the theory of MFH, i.e., there are food classes that can also be used as drugs.) (Jiang et al., 2015). Phytochemical studies have revealed that the chemical composition of this plant mainly includes iridoid and phenylpropanoid glycosides. Several compounds such as triterpenoids, flavonoids, and amino acids have been isolated from the plant (Qing et al., 2017; Chu et al., 2019). Currently, this species is used as a pulmonary demulcent and an emollient for curing sore throat, dire thirst, and constipation (Li and Zhang, 2000). Modern medicinal research shows that the extract of its ripe fruit can be commercially used as a supplement and as a sweetener in sugar-free health foods and drinks because the fruits contain sweet glycosides that are naturally low in calories (FX, 1996; Soejarto et al., 2019). In addition, the crude extracts and purified compounds from *S. grosvenorii* have been found to possess various biological activities including antioxidant, hypoglycemic, immunologic, anti-tussive and sputum-reducing, hepatoprotective, and antimicrobial activities (Liu et al., 2018; Chen et al., 2018; Abdel-Hamid et al., 2019; Nie et al., 2019; Zhang et al., 2019).Consequently, *S. grosvenorii* is a natural product with high development potential, which has increasingly been the focus of scientific research and commercial attention (Wang et al., 2010; Zhang and Li, 2011). The previous reviews focusing on the traditional uses, phytochemistry, pharmacology, toxicology, pharmacokinetics, and applications different aspects of *S. grosvenorii* are listed in **Table 1**.

**Table 1:** Overview of *S. grosvenorii* related reviews since 1983.

Topic	References
Traditional uses	Zeng 2009; Huang et al., 2012
Phytochemistry	Li HB et al., 2006; Xiao and Wang, 2006; Li et al., 2007; Lin et al., 2007; Liao et al., 2008; Zhang and Li, 2011; Chen and Jia, 2011; Li et al., 2014; Zhang et al., 2014
Pharmacology	Wang Q et al., 1999; Kamei and Sugiura, 2005; Xiao and Wang, 2006; Wang et al., 2010; Chen and Jia, 2011; Huang et al., 2012; Qin and Li, 2012; Long et al., 2012; Liu an Sun 2013; Li et al., 2014; Zhang et al., 2014; Zhang et al., 2017
Toxicology	Zhang and Li, 2011; Lin et al., 2012; Qin and Li, 2012
Pharmacokinetics	Wei et al., 2004
Applications	Chen et al., 1992; Kazama, 1999; Li HB et al., 2006; Zeng 2009; He et al., 2012; Pawar et al., 2013; Seki et al., 2017; Tan and Zeng, 2019

In this review, all of during the last 36 years (from 1983 to 2019) available information on *S. grosvenorii* was collected from the literary resources, such as PubMed, SciFinder Scholar, CNKI, TPL (www.theplantlist.org), Google Scholar, Baidu Scholar, and Web of Science. We aimed to provide comprehensive information on the botanical characterization, traditional uses and ethnopharmacology, food and nutritional values, chemical constituents, pharmacological effects, and toxicology of *S. grosvenorii*, and to assess valuable data for further applications to the *S. grosvenorii*, as well as suggestions regarding development direction.

## Botanical Characterization

**Figure 1:**
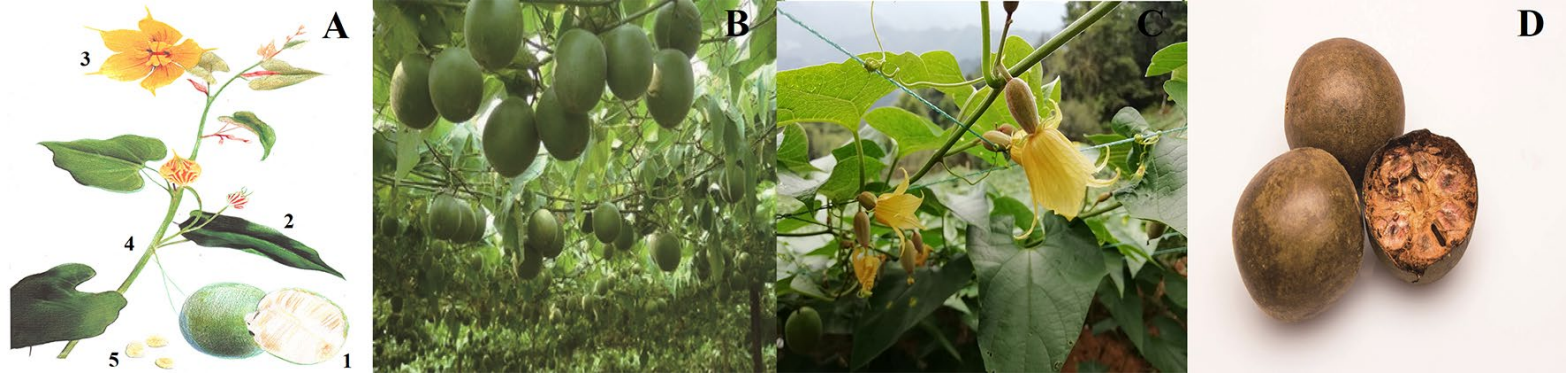
Images of *S. grosvenorii*. **(A)** Line drawing of *S. grosvenorii*: 1. fruit; 2. leaf; 3. flower; 4. stem; 5. seed. **(B)** and **(C)** Plant of *S. grosvenorii*. **(D)** The medicinal material of *S. grosvenorii*.

*S. grosvenorii* is a perennial vine with yellow-brown pubescence and black glandular scales. The roots are enlarged, fusiform, or subglobose (China national medicinal materials corporation, 1994); the stems and branches are slightly robust. The petioles are 3–10 cm; and the leaf blades are ovate-cordate, 12–23 × 5–17 cm, membranous, apex acuminate or long acuminate, sinus semicircular or broadly ovate-cordate. The male flowers are inflorescence racemose, 6−10-flowered; peduncle 7–13 cm; pedicels slender, 5–15 mm; calyx tube broadly campanulate, 4–5 × ca. 8 mm, usually with 3 membranous scales; segments triangular, ca. 4.5 × 3 mm, 3-veined, apex long acuminate; corolla yellow; segments oblong, 10–15 × 7–8 mm, 5-veined, apex acute; filaments puberulent, ca. 4 mm; anthers ca. 3 mm. The female flowers are solitary or 2–5 on a 6–8 mm peduncle; calyx and corolla as in male flowers but slightly larger; staminodes 2–2.5 mm; ovary oblong, 10–12 × 5-6 mm, densely yellow-brown velvety, base obtuse-rounded; style ca. 2.5 mm; stigmas 3, enlarged, ca. 1.5 mm. The fruits are globose or oblong, 6–11 × 4–8 cm, densely yellow-brown velvety and black glandular-scaly, ultimately glabrous. The seeds are numerous, pale yellow, broadly ovate, compressed, 15–18 × 10–12 mm, base obtuse-rounded, with 2-layered wings, wings sinuate (**Figure 1**) (Flora of China Editorial Board of the Chinese Academy of Sciences, 2004).

## Traditional Uses and Ethnopharmacology

As recorded in the Committee for the Pharmacopoeia of PR China (2015), *S. grosvenorii* has remarkable efficacy in treating coughs, sore throat, and excessive phlegm (Committee for the Pharmacopoeia of PR China, 2015). *Bencao Gangmu*, which is a famous monograph written on traditional Chinese medicine during the Ming Dynasty, is the first book to list the applications of this plant in China. The record of the use of *S. grosvenorii* as an expectorant for relieving sore throat, clearing heat, and moistening the lungs dates to 2,000 years ago. Furthermore, *S. grosvenorii* is used as a functional food because it contains several nutritious compounds, including mogrosides, trace elements, linoleic acid, vitamin C, and other unsaturated fatty acids (Li et al., 2014). Finally, *S. grosvenorii* is also used as a food source in East Asian countries, such as China, Japan, and South Korea. The traditional uses of *S. grosvenorii* in China are shown in **Table 2**.

**Table 2:** Traditional uses of *S. grosvenorii* in China.

Preparation name	Compositiona crude drug names (Latin names of original plants)^a^	Traditional uses	References
*Luohan Guo Decoction*	Siraitiae fructus (*Grosvenor momordica*) 10 g, Crataegi fructus (hawthorn) 10 g	Treating clearing heat and moistening lung	*Yi Shuo* (Song Dynasty, 1224 A.D.)
*Luohan Guoba Zheng Decoction*	Siraitiae fructus (*Grosvenor momordica*) 15 g, Longan arillus (*Arillus longan*) 15 g, Sauropi folium (*Sauropus spatulifolius Beille*) 50 g, Jujubae fructus (*Candied Jujube*) 10 g, Panacis quinquefolii radix (*Ginseng*) 20 g, Armeniacae semen amarum (*Prunus armeniaca* L.var.ansu Maxim) 20 g, Glehniae radix (*Glehnia littoralis* Fr. Schmidt ex Miq) 15 g	Curing phthisis and pertussis	*Ji Sheng Fang* (Song Dynasty, 1256 A.D.)
*Luohan Guorou Decoction*	Siraitiae fructus (*Grosvenor momordica*) 30–60 g, Pig lean meat 100 g	Curing phthisis and coughs	*Bencao Gangmu* (Ming Dynasty, 1578 A.D.)
*Luohan Guoyi Mu Decoction*	Siraitiae fructus (*Grosvenor momordica*) 15 g, Leonuri herba (*Leonurus japonicus Houtt*.) 10 g	Curing phthisis, coughs and irregular menstruation	*Yixue Xinwu* (Qing Dynasty, 1732 A.D.)
*Luohan Guoqing Fei Tea*	Siraitiae fructus (*Grosvenor momordica*) 20 g, Mume fructus (dark plum) 15 g, Lilii bulbus (*Lilium lancifolium* Thunb) 10 g, Desmodii styracifoliii herba (*Desmodium styracifolium* (Osb.) Merr.) 10 g, Apocyni veneti foliu (Apocynum venetum L) 10 g, Houttuyniae herba (*Houttuynia cordata* Thunb) 15 g	Treating clearing lung, resolving phlegm and antiviral	*Jifeng Puji Fang* (Qing Dynasty, 1828 A.D.)
*Luohan Guoshi Bing Decoction*	Siraitiae fructus (*Grosvenor momordica*) 30 g, Kaki calyx (dried persimmon) 15 g	Curing phthisis, pertussis and throat	*Huoren Fang* (Qing Dynasty, 1862A.D.)

a All the crude drug names in column 2 were identified according to the Chinese Pharmacopoeia, 2015, and the Latin names of the original plants were identified with TPL (www.theplantlist.org)

## Food and Nutritional Value

Commercially, *S. grosvenorii* is available as dried fruits, which are consumed after processing. In the 1990s, the China Food and Drug Administration (CFDA) approved the use of *S. grosvenorii* as a sweetener in foods (Cheng et al., 2015). In 1996, it was approved as a substitute for sweeteners in health foods for obesity and diabetes patients (Ren et al., 2009). As a new low-calorie, non-sugar sweetener, *S. grosvenorii* can be consumed as a health-promoting juice or as an additive or used to prepare sugar-free foods (Yang et al., 2016).

Several *S. grosvenorii* products are patented; one among such products is a health-promoting sugar-free *S. grosvenorii* beverage that preserves the medicinal effects of *S. grosvenorii*. This beverage is prepared by completely dissolving a powder formulation of *S. grosvenorii* in water and is suitable for treating diabetes (Zhang, 2011). Despite being a low-calorie product, it has high sweetness, making it an ideal new source of sugar for diabetes and obesity patients (Wu, 2011). Medicinal products developed from this plant include *Luohanguo Paoteng Tablet* and *Luohanguo yanhou Tablet*, which are used to alleviate pharynx discomfort (Jin et al., 1997; Jiang et al., 2011; Chen et al., 2015). *Luohanguo Lvcha Granules* are used to enhance immunity, and *Luohanguo Sydney Cream* and *Jinyinhua Luohanguo Lozenges* are used to cure sore throat (Zhang et al., 2017).

Stemoninine-mogroside V at mass ratios of 2:1 and 1:1 exerts effects in relieving cough and reducing sputum (Wu et al., 2017). The addition of concentrated *S. grosvenorii* juice to tobacco markedly reduces the harmful pulmonary effects of smoking, reduces pharynx discomfort, and promotes smoking cessation (Li D et al., 2006).

In addition, foods developed using *S. grosvenorii* include *Luohanguo*-fermented wine, *Luohanguo* cake, *Luohanguo* preserved fruit, and *Luohanguo* fruit compound beverage (Li D et al., 2006). Moreover, there are many patented products of *S. grosvenorii* that find applications in the cosmetic industry due to their skin whitening and moisturizing effects (Zhang and Zhang, 2011; Yang, 2016).

## Chemical Constituents

Based on the existing literature, more than 100 compounds have been isolated from *S. grosvenorii*, including at least 46 triterpenoids, 7 flavonoids, 19 amino acids, and 2 polysaccharides.

### Triterpenoids

**Figure 2:**
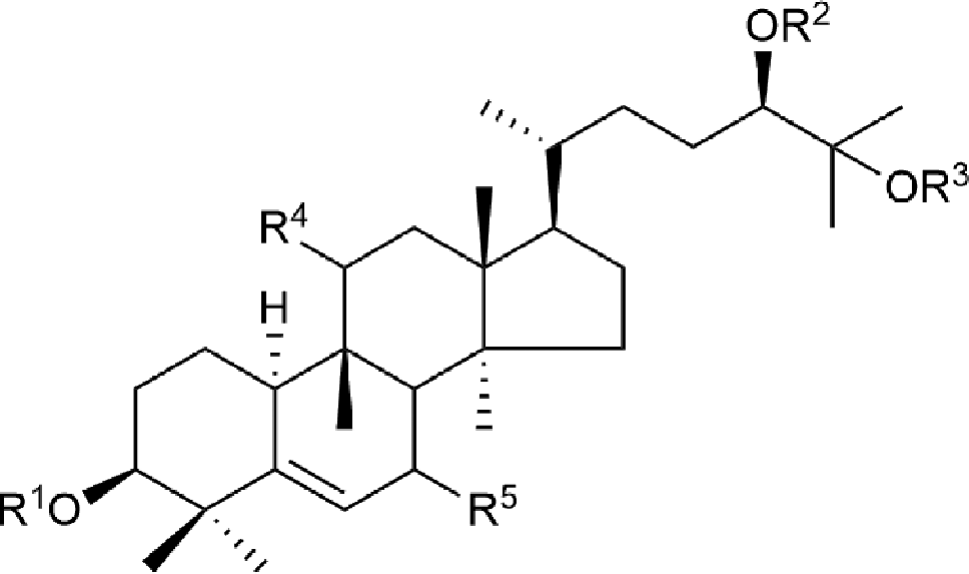
The skeletal structures of triterpenoid from *S. grosvenorii*.

**Figure 3:**
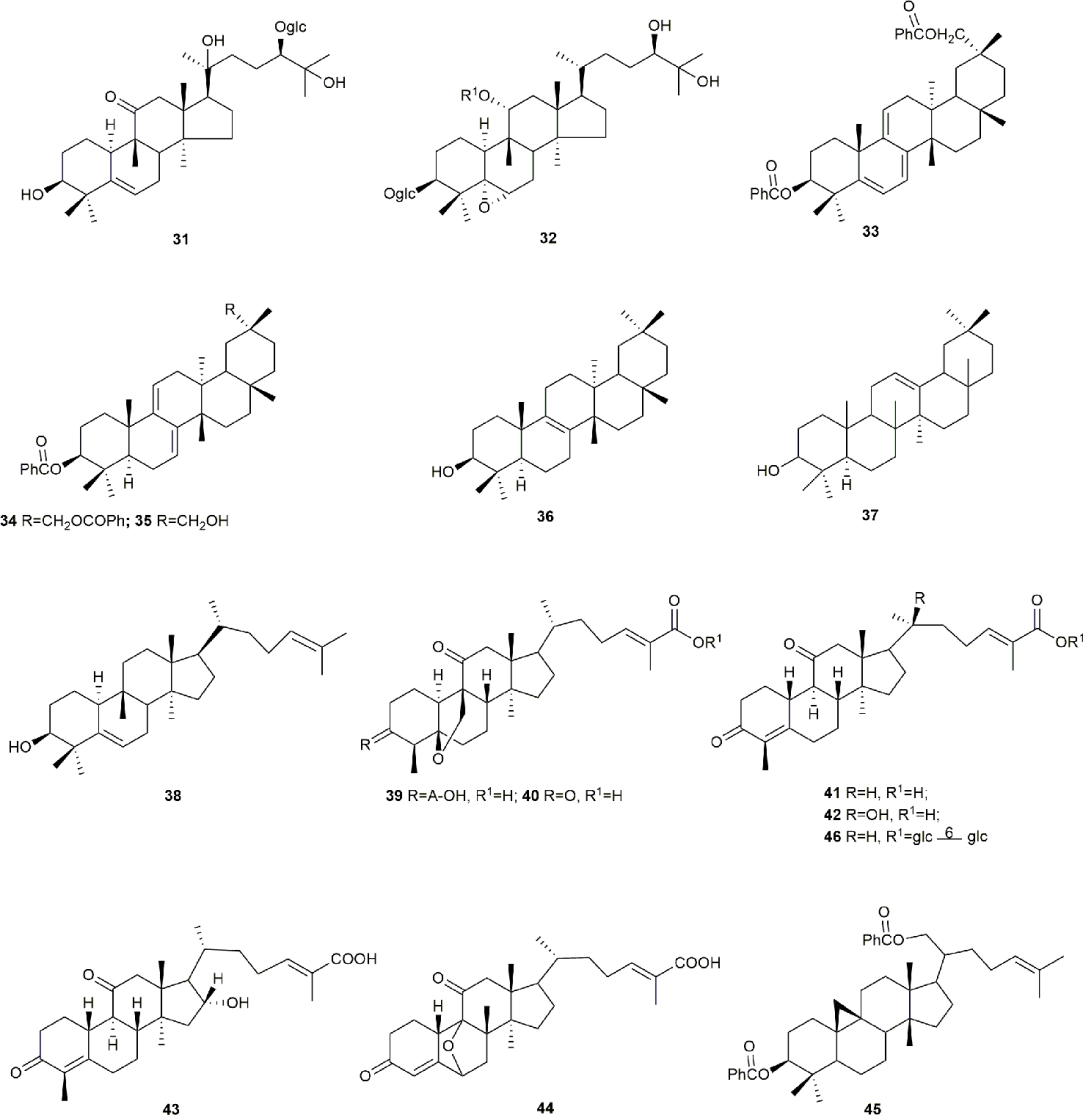
The structures of the triterpenoid compounds isolated from *S. grosvenorii*.

Cucurbitane glycosides are the active ingredients of *S. grosvenorii* fruits. Mogrosides IV, V, and VI were successfully isolated from *S. grosvenorii* fruits in 1983 (Takemoto et al., 1983a; Takemoto et al., 1983b). Simultaneously, more than 30 similar compounds have been obtained from the fruits, and these compounds have the mogrolaglycone structure, [10-cucurbit-5-ene-3, 11, 24(R), 25-tetraol], with different numbers of glucose units attached (**Table 3**, **Figures 2** and **3**).

**Table 3:** Triterpenoid compounds isolated from *S. grosvenorii*.

No.	Compound name	R^1^	R^2^	R^3^	R^4^	R^5^	Ref.
1	Mogrol	H	H	H	a-OH	H_2_	(Ren et al., 2009; Cheng et al., 2015; Yang et al., 2016)
2	Mogroside IA (Mogroside, A1)	H	glc	H	H_2_	H_2_	(Zhang, 2011; Yang et al., 2016)
3	Mogroside IE1	glc	H	H	a-OH	H_2_	(Zhang, 2011; Yang et al., 2016)
4	Mogroside IIA1	H	glc-6-glc	H	a-OH	H_2_	(Yang et al., 2016; He et al., 2017)
5	Mogroside IIA2	glc-6-glc	H	H	a-OH	H_2_	(Yang et al., 2016)
6	Mogroside IIE	glc	glc	H	a-OH	H_2_	(Xu et al., 1992; Wei et al., 2004; Li D et al., 2006; Song et al., 2009; Zhang, 2011; Tan et al., 2014; Yang et al., 2016)
7	Mogroside IIIA1	H	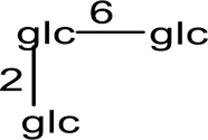	H	a-OH	H_2_	(Takemoto et al., 1983b; Li et al., 2011)
8	Mogroside IIIA2	glc-6-glc	glc	H	a-OH	H_2_	(Wei et al., 2004; Yang et al., 2016)
9	Mogroside IIIE	glc	glc-2-glc	H	a-OH	H_2_	(Zhang, 2011; Yang et al., 2016)
10	Mogroside IVA	glc-6-glc	glc-6-glc	H	a-OH	H_2_	(Li D et al., 2006; Jiang et al., 2011; Zhang, 2011; Chen et al., 2015; Yang et al., 2016)
11	Mogroside IVE	glc-6-glc	glc-2-glc	H	a-OH	H_2_	(Wei et al., 2004; Jiang et al., 2011; Zhang, 2011; Yang et al., 2016)
12	Mogroside IIB	glc	H	glc	a-OH	H_2_	(He et al., 2017)
13	Mogroside III	glc	glc-6-glc	H	a-OH	H_2_	(Wei et al., 2004; Li D et al., 2006; Zhang, 2011; Tan et al., 2014)
14	Mogroside V	glc-6-glc		H	a-OH	H_2_	(Wei et al., 2004; Jiang et al., 2011; Zhang, 2011; Chen et al., 2015; Yang et al., 2016)
15	Mogroside VI	glc-6-glc		H	a-OH	H_2_	(Ren et al., 2009)
16	Siamenoside I	glc		H	a-OH	H_2_	(Jia and Yang, 2009; Jiang et al., 2011; Zhang, 2011; Chen et al., 2015)
17	Neomogroside	glc-6-glc-2-glc		H	a-OH	H_2_	(Si et al., 1996; Wei et al., 2004)
18	Isomogroside V	glc-4-glc		H	a-OH	H_2_	(Jiang et al., 2011)
19	Grosmomoside I	glc		H	a-OH	H_2_	(Jin et al., 1997; Zheng et al., 2011)
20	11-Oxomogrol	H	H	H	O	H_2_	(Zhang et al., 2017)
21	11-Oxomogroside IA1	H	glc	H	O	H_2_	(Li D et al., 2006; Zhang et al., 2017)
22	11-Oxomogroside IE 1	glc	H	H	O	H_2_	(Zhang et al., 2017)
23	11-Oxomogroside IIA1	H	glc-6-glc	H	O	H_2_	(Ukiya et al., 2002)
24	11-Oxomogroside IIE	glc	glc	H	O	H_2_	(Li D et al, 2006)
25	11-Oxomogroside III	glc	glc-6-glc	H	O	H_2_	(Li et al., 2010)
26	11-Oxomogroside IVA	glc-6-glc	glc-6-glc	H	O	H_2_	(Akihisa et al., 2007; He et al., 2017)
27	11-Oxomogroside V	glc-6-glc		H	O	H_2_	(Matsumoto et al., 1990; Zhang, 2011; Tan et al., 2014)
28	7-Oxomogroside IIE	glc	glc	H	a-OH	O	(He et al., 2017)
29	7-Oxomogroside V	glc-6-glc		H	a-OH	O	(He et al., 2017)
30	11-DeoxymogrosideIII	glc	glc-6-glc	H	H_2_	H_2_	(Mi et al., 2015; He et al., 2017)

### Flavonoids

**Figure 4:**
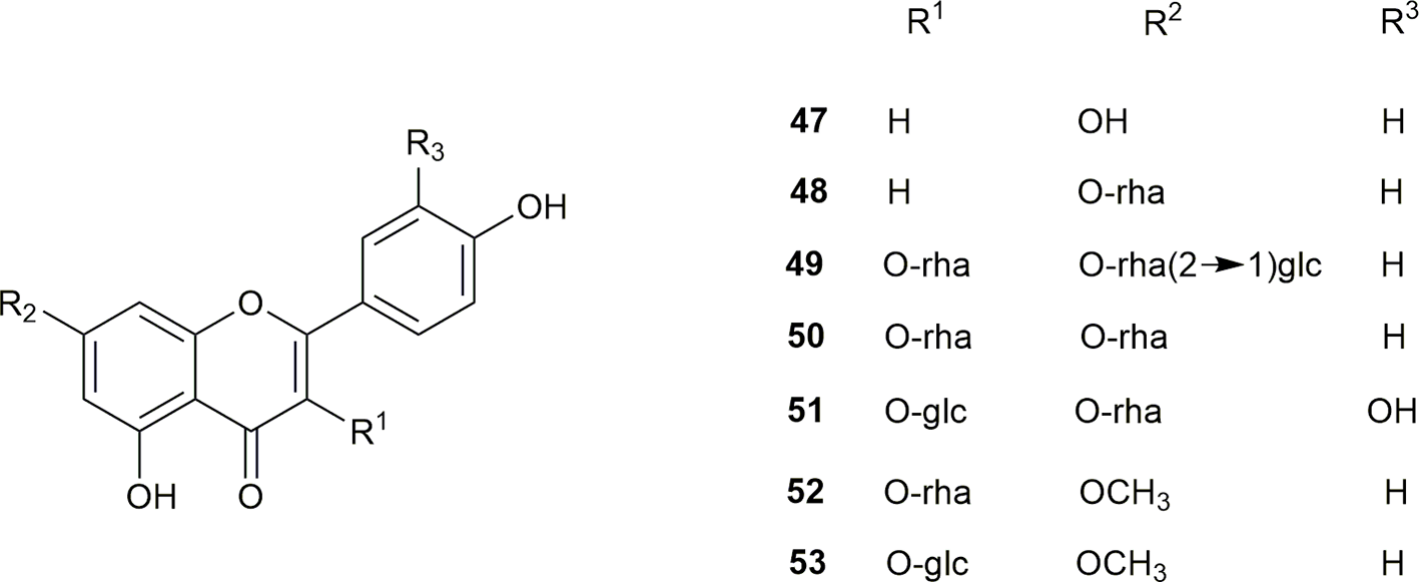
The structures of flavonoids from *S. grosvenorii*.

Seven flavonoids have been isolated from the flowers of *S. grosvenorii*, namely, kaempferol (**47**), kaempferol 7-*O*-L-rhamnopyranoside (**48**), kaempferol 3-*O*-L-rhamnopyranoside-7-*O*-[*β*-D-glucose-based-(1-2)-*α*-L-rhamnoside] (**49**), 3-*O*-L-rhamnopyranoside (**52**), and 3-*O*-D-glucopyranoside (**53**). Kaempferol 3,7-di-*O*-L-rhamnopyranoside (kaempferitrin) (**50**) and quercetin-3-*O*-D-glucopyranoside-7-*O*-L-rhamnopyranoside (**51**) were isolated from the leaves of *S. grosvenorii* (Li et al., 2003; Li et al., 2007; Liao et al., 2008). The flavonoid structures are shown in **Figure 4**.

### Others

**Figure 5:**
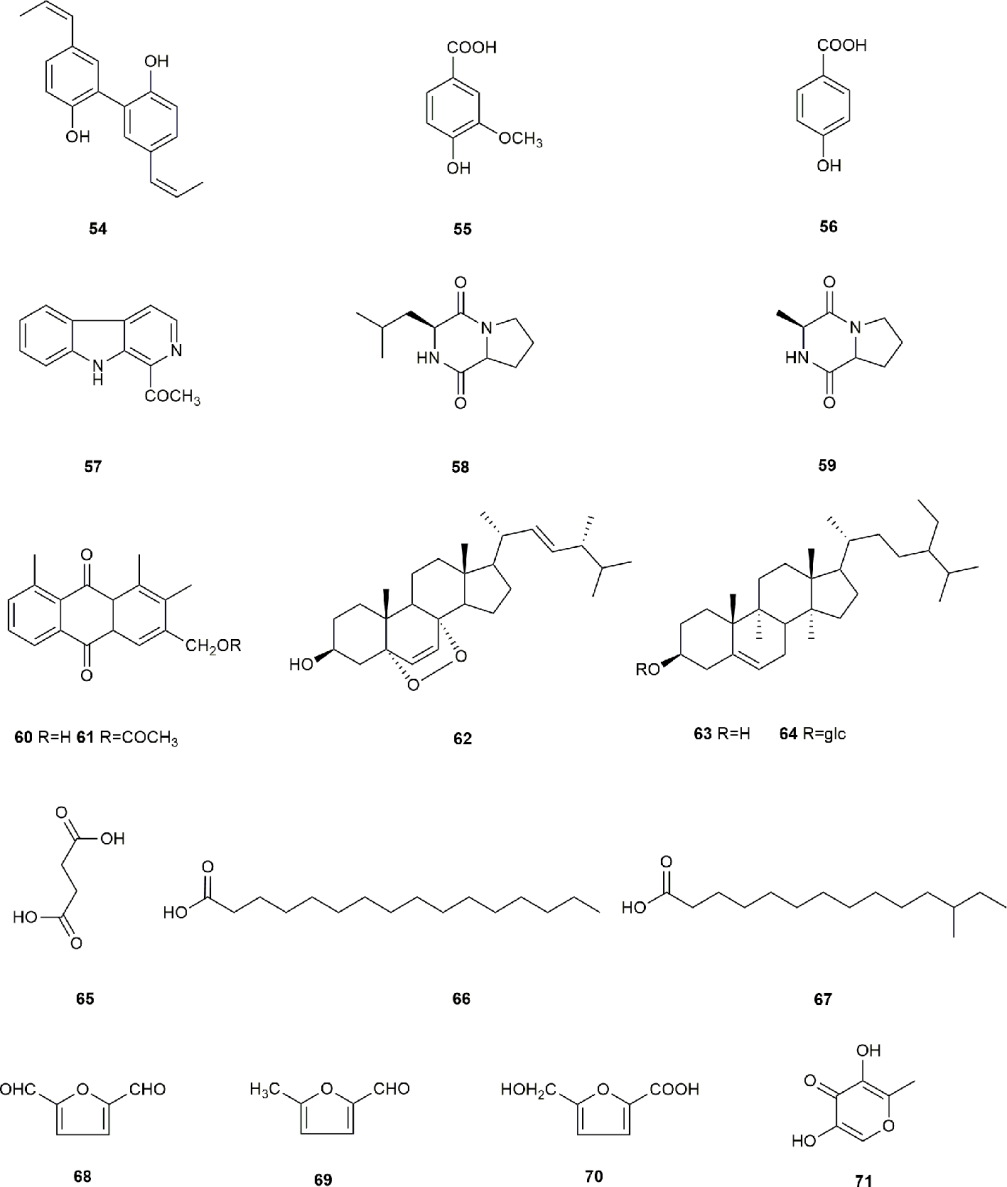
The structures of other compounds from *S. grosvenorii*.

Other types of compounds have also been identified from *S. grosvenorii*, such as magnolol (**54**), vanillic acid (**55**), *p*-hydroxybenzylic acid (**56**), 1-acetyl-β-carboline (**57**), *cyclo*-(leu-pro) (**58**), *cyclo*-(ala-pro) (**59**), aloe emodin (**60**), aloe-emodin acetate (**61**), 5, 8-epidioxy-24(*R*)-methylcholesta-6, 22-dien-3β-ol (**62**), β-sitosterol (**63**), daucosterol (**64**), succinic acid (**65**), *n*-hexadecanoic acid (**66**), 12-methyltetradecanoic acid (**67**), 5-hydroxymethylfurfural (**68**), 5, 5'-oxydimethylenebis(2-furfural) (**69**), 5-(hydroxymethyl)-furoic acid (**70**), and 5-hydroxymaltol (**71**) (Chen et al., 2004; Chen QB et al., 2006; Yao et al., 2008). The structures of these compounds are shown in **Figure 5**.

## Pharmacological Effects

### Antioxidant Effects

Hossen have reported that the *S. grosvenorii* extract prevents the generation of superoxide anion and inhibits histamine release from the mast cells and histamine-induced nose scratching behavior in ICR mice (Hossen and Sun, 2006). These functions are related to the antioxidant activity of *S. grosvenorii*. The scavenging capacity of mogrosides on reactive oxygen species (ROS) was studied by the chemiluminescence method. The results showed that crude extracts of *s. grosvenorii* have high scavenging capacity and that the antioxidant effect was almost the same as that of ascorbic acid (V_C_) (Zhu et al., 2015). Mogroside extract effectively eliminates free radicals, reduces the incidence of hemolysis of Fe^2+^, and alleviates hydrogen peroxide induced-oxidative damage to hepatic tissues. Hydroxyl radicals and superoxide anion radicals are involved in eliminating free radicals; with increasing concentration of mogroside extract, the scavenging effect gradually increases, thereby exhibiting a dose-effect relationship (Lan et al., 2018).

In a study assessing the *in vitro* antioxidant activity, it was suggested that the sweet cucurbitane glycosides mogroside V (**14**) and 11-oxo-mogroside V (**27**) from the fruits of *S. grosvenorii* have remarkable ROS scavenging ability, with different antioxidant effects in different systems. Furthermore, as compared to mogroside V (EC_50_ = 16.52 mg/ml), 11-oxo-mogroside V (EC_50_ = 4.79 mg/ml) exhibited stronger scavenging activity against superoxide anions and hydrogen peroxide, and stronger inhibitory effect on DNA damage, but weaker scavenging effect on hydroxyl radical. Therefore, these two compounds could be effective as free radical-scavenging agents (Chen et al., 2007).

Exhaustive exercise causes increased oxygen consumption, hypoxia in local tissues, and accumulation of toxic metabolites, which affects the oxidative function of the mitochondria. A study was conducted to establish an animal model of exhaustive exercise and to explore the effects of flavonoids from the leaves of *S. grosvenorii* on the myocardial mitochondria of rats subjected to vigorous exercise. The flavonoids effectively eliminate the endogenous capacity of production of ROS caused by vigorous exercise in the rats. Thus, it was revealed that flavonoids from the leaves of *S. grosvenorii* can promote blood circulation and exert a strong antioxidant activity (Xie, 2018).

Four types of extracts of *S. grosvenorii* have been successfully prepared and their antioxidant effects have been determined. The results showed that the four extracts had antioxidant and radical scavenging activities and that the strength of their antioxidant effects follows the following order: ethyl acetate extract > water extract > methanol extract > ethanol extract. Thus, this evidence indicates that *S. grosvenorii* might be valuable as a health medicine and food (Li et al., 2008).

### Hypoglycemic Effects

It has been revealed that mogroside is the main active ingredient responsible for the hypoglycemic effect of *S. grosvenorii*. The extract of *S. grosvenorii* alleviates alloxan-induced damage and repairs β cells to alleviate symptoms in diabetic mice (Qi et al., 2003). Studies have shown that the *S. grosvenorii* powder and extracts have no effects on blood glucose level and glucose tolerance in normal mice; however, a significant hypoglycemic effect was observed in alloxan-induced diabetic mice. In contrast, serum levels of triglycerides and cholesterol abnormally increased and the serum high-density lipoprotein cholesterol and blood lipid level increased; blood lipid level tended to be normal to prevent lipid metabolism disorders caused by diabetes. In addition, the *S. grosvenorii* extract can induce repair of islet β cells to relieve the symptoms of diabetes in mice (Qi et al., 2003). In addition, active substances of *S. grosvenorii* responsible for the hypoglycemic effect include flavonoids and polysaccharides. Currently, the main hypoglycemic mechanism of *S. grosvenorii* involves the repair of pancreatic injury, promotion of insulin secretion, scavenging of free radicals and anti-lipid peroxidation, and inhibition of intestinal α-glucosidase activity (Wan et al., 2016).

In a study by Suzuki, rats with type II diabetes were fed 40% mogroside extract for 13 weeks. Consequently, it was observed that insulin response in the rats was improved; blood sugar levels, urine amount, and protein levels were reduced; and diabetic complications were prevented. The *S. grosvenorii* extract and mogroside V have been further studied in rat β cell line RIN-5F. The results showed that *S. grosvenorii* extract and mogroside V can significantly promote insulin secretion and regulate blood sugar levels (Suzuki et al., 2007). In addition, the *S. grosvenorii* extract not only reduces blood sugar level but also prevents oxidative stress-related complications in alloxan-induced diabetic mice. The extract reduces the development of diabetes and vascular endothelial injury as well as the incidence of diabetic nephropathy (Zhang et al., 2006).

The mogroside extract also significantly decreases the activity of heme oxygenase-1 (HO-1), manganese superoxide dismutase (MnSOD), and glutathione peroxidase; inhibits the mRNA expression of HO-1 and MnSOD; increases serum HDL-C level; and regulates the activity of antioxidant enzymes in the liver, thereby decreasing the symptoms of diabetic nephropathy in mice with diabetes. The mogroside extract effectively improves clinical symptoms, increases insulin secretion, and decreases the pathological damage of islets in insulin-dependent diabetic mice (Wang Q et al., 1999).

### Immunologic Effects

*S. grosvenorii* polysaccharides (SGP) significantly increased the weight of mouse thymus, spleen, and other immune organs; percent of phagocytic cells; level of serum hemolysin; transformation rate of lymphocytes; and function of the immune system. It has been reported that oral administration of SPG at 1,200 and 100 Mg/Kg increases serum hemolysin (IgM) level, lymphocyte transformation rate, and thymus and spleen indexes. The results indicated that Sgp obviously enhances humoral and nonspecific immunity. intraperitoneal injection of Sgp to mice not only significantly increases thymus and spleen indexes but also improves NO0 and H_2_O_2_ levels, SOD activity, and hydroxyl radical scavenging capacity. Furthermore, SGP affects immune functions by adjusting the level of free radicals (Wang et al., 1994).

To study the effects of SGP on the immune function of immunosuppressive mice, experimental mice were randomly divided into three groups, *viz.* normal group, model group, and treated groups (25, 50, and 100 mg/kg). A mouse model of immunosuppression was established *via* the intragastric administration of cyclophosphamide (20 mg/kg) for 14 days. The effect of SGP on the immune function of the immunosuppressed mice was investigated by examining the levels of immunoglobulin (Ig)G, IgM, interleukin (IL)-2, IL-4, IL-6, and tumor necrosis factor (TNF)-α. The results indicated that SGP significantly improves the immune function of immunocompromised mice (Zhang et al., 2018).

### Anti-Tussive and Sputum-Reducing Effects

Chen Y et al. (2006) studied the antitussive activities of mogrosides by testing the coughing frequency induced by ammonium hydroxide in mice and the expectorant activities by testing the amount of phlegm secreted in mice. The oral dose of mogrosides was higher than 15 g/kg that retrained mice's cough resulted in ammonia water, increased the density of phenol red secreted from windpipe of mice. According to the sequential method for the principle of median effective dose, the antitussive effect of *S. grosvenorii* was observed and the median time to observe antitussive effect induced by ammonia water in mice was calculated. Tang et al. (2015) investigated the effects of different factors on the antitussive effect of *S. grosvenorii*. The results showed that *S. grosvenorii* in different habitats had different antitussive effects, and the fruit growth period and commercial specifications influenced the antitussive effect of *S. grosvenorii*.

Research has shown the sputum-reducing effect of the *S. grosvenorii* extracts by increasing the excretion of phenol red from the rat trachea, as well as the excretion of phlegm from the rat trachea (Liu and Sun, 2013). It was reported in a rat model study that mogrosides at doses of 400 and 800 mg/kg could significantly increase the excreted amount of phlegm dose-dependently. And the sputum-reducing effect of 800 mg/kg dosage group was equal to that of ammonium chloride positive group (Chen Y et al., 2006). Furthermore, in order to investigate the relationship between expectorant efficacy and active components of *S. grosvenorii*, the phenol red secretion of trachea in mice and the HPLC fingerprint of *S. grosvenorii* extract were determined (Wang et al., 2017). Grey relational analysis was applied to confirm the contribution degree of each common peak from HPLC fingerprints for expectoration efficacy, while partial least-squares regression was utilized to confirm either positive or negative relationship, and to identify the contribution degree of *S. grosvenorii* extract. The results indicated that expectorant efficacy of *S. grosvenorii* is contributed by a combined action of multi-components rather than one component. And among them, oxomogroside V and mogroside V (**14**) have sputum-reducing effect and high contribution degree (Wang et al., 2017).

### Hepatoprotective Effects

Mogroside V (**14**) is the major saponin in *S. grosvenorii*. Gang evaluated the effect of mogrosides (the main constituents of mogroside V, **14**) on carbon tetrachloride-induced liver injury in Kunming mice, and *Bacillus* Calmette–Guerin (BCG)- and lipopolysaccharide (LPS)-induced liver injury. Mogrosides exert no effect on enzyme activities in normal liver but reduce the serum levels of alanine aminotransferase (ALT), aspartate transaminase (AST), and malondialdehyde (MDA). The activity of SOD was significantly reduced in the liver tissue, proving that mogrosides exert a protective effect in mice with acute liver injury (Xiao and Wang, 2008). In another study, it was revealed that the *S. grosvenorii* extract significantly improved the swimming time of mice. It effectively increases the activity of SOD and glutathione peroxidase (GSH-Px), removes blood lactic acid (BLA), enhances the body's antioxidant capacity, promotes hemoglobin synthesis, and reduces the amount of BLA generated (Wang T et al., 1999). Thus, *S. grosvenorii* extract was shown to exert a significant protective effect against liver injury.

### Antimicrobial Effects

The leaf, fruit, and stem extracts of *S. grosvenorii* possess strong inhibitory effect against *Pseudomonas aeruginosa*, *Escherichia coli*, and *Streptococcus mutans*. To examine the antibacterial effect of *S. grosvenorii* fruits, crude extracts of dried *S. grosvenorii* fruits were prepared, and 50 components were separated by high-performance liquid chromatography. The results revealed that compounds 18−19 and 34−35 have strong inhibitory activity, whereas the other compounds did not exhibit any or exhibited only weak activity. In addition, mogroside V had no antibacterial activity (Zhou and Huang, 2008). Qi et al. (2006) studied the antimicrobial effect of *S. grosvenorii* ethanol extract on *P. aeruginosa*, *Staphylococcus aureus*, and *Candida albicans*. The inhibitory rate of the extract against these different strains was measured using the dichotomy method. It was found that the ethanol extracts of leaves and stems of *S. grosvenorii* have antimicrobial activity, with inhibition values of 70.2% against *P. aeruginosa* and 50% or less against *S. aureus* and *C. albicans*.

### Other Effects

Besides the pharmacological effects mentioned above, *S. grosvenorii* also possesses other activities, such as anticancer and anti-fatigue effects. In a previous study, dimethyl benzanthracene was used as an initiator and 12-*O*-tetradecanoyl-phorbol-13-acetate as an accelerator in a two-stage mouse skin carcinogenicity assay. The results showed that mogroside V (**14**) has a good inhibitory effect (Suzuki et al., 2007). In addition, the tubers of *S. grosvenorii* have been shown to possess significant anticancer activity *in vitro* (Takasaki et al., 2003). It is found that *S. grosvenorii* extract inhibited the expression of Cyp1a1, which plays a role in inhibiting liver cancer (Matsumoto et al., 2009). Furthermore, Zhang et al. studied the anti-fatigue effect of *S. grosvenorii* extract in 144 male ICR mice, and the results showed that liver and muscle concentrations of glycogen in the low-, medium-, and high-dose *S. grosvenorii* extract group were significantly higher than those in the control group (*p* < 0.05). It has been proven that *S. grosvenorii* fruit extract has obvious anti-fatigue effect in mice with exercise fatigue (Zhang et al., 2017).

## Toxicology

Currently, the toxicity of *S. grosvenorii* is considered low and its use as MFH species is safe (Zhang and Li, 2011). Qin et al. investigated the safety of PureLo, a non-caloric powdered concentrate of *S. grosvenorii*, which derives its sweetening properties from triterpene glycosides called mogrosides. Male and female dogs were administered 3,000 mg/kg bw/day PureLo for either 28 or 90 days. Body weight, blood chemistry, food consumption, urinalysis, organ weight, and other indexes were evaluated to analyze the toxicity of PureLo. The results showed no changes in body weight, organ weight, and food consumption. There were no significant effects on blood chemistry analysis and urinalysis results. The results indicated that PureLo does not induce any organ or systemic toxicity (Qin et al., 2006). In another study, 20 Kunming mice were administered *S. grosvenorii* extract (7,200 mg/kg bw) for 12 h and then observed for one week. No changes in organ morphology and no short-term toxicity were observed. Furthermore, *Salmonella typhimurium* TA97, TA98, TA100, and TA102 were used in Ames assay with mogroside (50, 5, 0.5, 0.05, and 0.002 mg/ml) in the presence or absence of S9 metabolic activator. The result showed that mogroside exerts no genotoxic effect (Hussain et al., 1990). Marone et al. conducted an experiment on Sprague-Dawley rats to evaluate the safety of PureLo. Twenty rats (10/sex/group) were fed PureLo (0, 10,000, 30,000, or 100,000 ppm) for 28 days (OECD, Redbook 2000). The results showed no significant adverse effects. There were no differences in body weight and feed efficiency, but there was a decrease in bilirubin level and an increase in total protein content. Overall, the no observed adverse effect level (NOAEL) of PureLo in the diet was 100,000 ppm, which is equivalent to 7.07 and 7.48 g/kg bw/day for male and female rats, respectively (Marone et al., 2008).

## Conclusions and Future Perspectives

Numerous studies have been conducted to investigate the pharmacological activities of *S. grosvenorii*, including its antioxidant, hepatoprotective, hypoglycemic, immunologic, and anti-inflammatory activities, etc. In this review, the research progress of the traditional uses and pharmacological activities of *S. grosvenorii* during the last 36 years is summarized. We believe that these pharmacological still deserve further research. Information on the mechanism of action of this plant will help its development as a new health food and a novel therapeutic agent in the future.

Only a few studies have been conducted on its pharmacokinetics and clinical applications. Among the several classes of biologically active compounds identified in *S. grosvenorii*, mogrosides are assumed to be the main active components responsible for most of the pharmacological actions of *S. grosvenorii*. However, the biological effects of the other chemical components as well as the interactions of mogrosides with the other compounds cannot be ruled out. Further studies are needed to elucidate the complex pharmacological actions and the complete phytochemical profile of the plant (Li et al., 2014). Moreover, clinical studies should also be conducted to thoroughly evaluate the therapeutic effects, adverse effects, and toxicity of *S. grosvenorii*.

*S. grosvenorii* is an important traditional Chinese medicinal herb and commodity. With increasing demand from international markets, the need for high-quality *S. grosvenorii* has increased to satisfy the higher standards expected (Meng and Liao, 2018). However, there are no uniform standard systems and quality grade standards for the cultivation and management of *S. grosvenorii* (Wang et al., 2010). Li determined the content of Cu and Cd in the fruits of four *S. grosvenorii*-producing areas by flame atomic absorption spectrometry, and the results showed that the Cd content seriously exceeded the standard (Li F et al., 2006). Because heavy metal pollution is a threat to human health, we should focus on establishing a GAP standard in the management of Chinese medicinal herbs. This can assist in the sustainable production and management of *S. grosvenorii* and further improve its efficacy and safety.

In a word, phytochemical and pharmacological studies of *S. grosvenorii* have received great interest, and an increasing number of extracts and active compounds have been isolated that have demonstrated antioxidant, hypoglycemic, immunologic, anti-tussive and sputum-reducing, hepatoprotective, and antimicrobial activities. However, validating the correlations of the chemical composition and pharmacological effects should be carried out further, and pharmacokinetics and clinical applications of *S. grosvenorii* also should be studied systematically. Meanwhile, the market demand and quality control also should be highly regarded.

## Author Contributions

ML and JW conceived the review. XG, NC, KR, and LZ wrote the manuscript. JJ, and YL collected the literatures. KW and ML edited the manuscript. All authors read and approved the final version of the manuscript.

## Funding

This work was supported by National Natural Science Foundation of China (No. 81874336); Natural Science Foundation of Inner Mongolia Autonomous Region (No.2018ZD13); China Agriculture Research System (No.CARS-21); the Fourth National Traditional Chinese Medicine Resources Survey Project; Science and Technology Innovation Guidance Project, Inner Mongolia; the TCM (Traditional Chinese Medicine) Standardization Project From State Administration of TCM (No.ZYY-2017-069); Inner Mongolia autonomous region science and technology innovation leading project.

## Conflict of Interest

The authors declare that the research was conducted in the absence of any commercial or financial relationships that could be construed as a potential conflict of interest.
