# Corrigendum: The Fruits of *Siraitia grosvenorii*: A Review of a Chinese Food-Medicine

**DOI:** 10.3389/fphar.2019.01627

**Published:** 2020-01-30

**Authors:** Xue Gong, Namuhan Chen, Kai Ren, Junying Jia, Kunhua Wei, Le Zhang, Ying Lv, Jianhua Wang, Minhui Li

**Affiliations:** ^1^ Department of Pharmacy, Baotou Medical College, Baotou, China; ^2^ Guangxi Key Laboratory of Medicinal Resources Protection and Genetic Improvement, Guangxi Botanical Garden of Medicinal Plants, Nanning, China; ^3^ Agricultural College, Inner Mongolia University for Nationalities, Tongliao, China; ^4^ Pharmaceutical Laboratory, Inner Mongolia Autonomous Region Academy of Chinese Medicine, Hohhot, China; ^5^ Department of Technology, Chifeng Institute for Drug Control, Chifeng, China; ^6^ Department of Pharmacy, Inner Mongolia Medical University, Hohhot, China; ^7^ Inner Mongolia Key Laboratory of Characteristic Geoherbs Resources Protection and Utilization, Baotou Medical College, Baotou, China

**Keywords:** *Siraitia grosvenorii*, ethnopharmacology, food and nutritional value, chemical constituent, antioxidant

In the published article, there was an error regarding the affiliation for “Kunhua Wei.” As well as having affiliation “4,” they should also have “Guangxi Key Laboratory of Medicinal Resources Protection and Genetic Improvement, Guangxi Botanical Garden of Medicinal Plants, Nanning, China”.

The authors apologize for this error and state that this does not change the scientific conclusions of the article in any way. The original article has been updated.

